# N-Myc overexpression increases cisplatin resistance in neuroblastoma via deregulation of mitochondrial dynamics

**DOI:** 10.1038/cddiscovery.2016.82

**Published:** 2016-12-12

**Authors:** Gabriella Casinelli, Jeff LaRosa, Manika Sharma, Edward Cherok, Swati Banerjee, Maria Branca, Lia Edmunds, Yudong Wang, Sunder Sims-Lucas, Luke Churley, Samantha Kelly, Ming Sun, Donna Stolz, J Anthony Graves

**Affiliations:** 1 Department of Pediatrics/ Division of Hematology-Oncology, University of Pittsburgh, Pittsburgh, PA, USA; 2 Department of Pediatrics/ Division of Newborn Medicine, University of Pittsburgh, Pittsburgh, PA, USA; 3 Department of Pediatric Surgery, University of Pittsburgh, Pittsburgh, PA, USA; 4 Department of Microbiology and Molecular Genetics, University of Pittsburgh, Pittsburgh, PA, USA; 5 Department of Pediatrics/ Division of Medical Genetics, University of Pittsburgh, Pittsburgh, PA, USA; 6 Department of Pediatrics/ Division of Nephrology, University of Pittsburgh, Pittsburgh, PA, USA; 7 Department of Cell Biology/Center of Biological Imaging, University of Pittsburgh, Pittsburgh, PA, USA

## Abstract

N-Myc is a global transcription factor that regulates the expression of genes involved in a number of essential cellular processes including: ribosome biogenesis, cell cycle and apoptosis. Upon deregulation, N-Myc can drive pathologic expression of many of these genes, which ultimately defines its oncogenic potential. Overexpression of N-Myc has been demonstrated to contribute to tumorigenesis, most notably for the pediatric tumor, neuroblastoma. Herein, we provide evidence that deregulated N-Myc alters the expression of proteins involved in mitochondrial dynamics. We found that N-Myc overexpression leads to increased fusion of the mitochondrial reticulum secondary to changes in protein expression due to aberrant transcriptional and post-translational regulation. We believe the structural changes in the mitochondrial network in response to N-Myc amplification in neuroblastoma contributes to two important aspects of tumor development and maintenance—bioenergetic alterations and apoptotic resistance. Specifically, we found that N-Myc overexpressing cells are resistant to programmed cell death in response to exposure to low doses of cisplatin, and demonstrated that this was dependent on increased mitochondrial fusion. We speculate that these changes in mitochondrial structure and function may contribute significantly to the aggressive clinical ph9enotype of N-Myc amplified neuroblastoma.

## Introduction

Neuroblastoma accounts for 7% of malignancies from birth to 14 years of age^1,2^ and 12% of cancer deaths in children.^3^ Over 40% of neuroblastomas are considered high risk^4^ and >50% of patients survive.^5^ One important factor in defining high-risk disease is amplification of the *N-MYC* gene.^1,6,7^ Stage IV disease with *N-MYC* amplification has a 25–30% 5-year survival rate.^1^ The *N-MYC* gene has been estimated to be amplified in 15–25% of neuroblastomas,^8,9^ yet the mechanisms by which it drives pathophysiology remain elusive.

The *N-MYC* gene product (N-Myc) is a global transcription factor that regulates genes involved in growth and proliferation.^8,10,11^ Unlike its ubiquitous sister protein c-Myc,^12–14^ N-Myc displays a restricted pattern of expression; it is essential during embryonic neuronal development in the development of lungs, mesonephric tubules, neuroepithelium, and sensory ganglia, GI tract, and the heart.^15,16^ Once overexpressed, N-Myc possesses the oncogenic potential of c-Myc,^17,18^ but given its restricted expression, has been implicated in a smaller subset of tumors, including: retinoblastoma,^19^ small cell lung carcinoma,^20^ and neuroblastoma.^21,22^

In mammalian cells, normal c-Myc expression is required for proper mitochondrial biogenesis,^23–26^ including mitochondrial dynamics.^24^ Mitochondrial dynamics are fission and fusion events that dictate changes in size, shape, and cellular distribution of the organelle.^27–29^ c-Myc overexpression increased the levels of proteins involved in mitochondrial dynamics as much as two- to threefold,^24^ which resulted in increased mitochondrial fusion. As a more fused mitochondrial reticulum has been shown to increase oxidative phosphorylation (OXPHOS), it is believed that c-Myc overexpression increased ATP production by enhancing mitochondrial fusion. Given their functional similarities, we hypothesized that overexpression of N-Myc would deregulate mitochondrial biogenesis as well. In this study, we demonstrated that N-Myc overexpression in neuroblastoma increased mitochondrial biogenesis by the upregulation of mitochondrial fusion; however, this did not increase OXPHOS. Instead, this increase in fusion resulted in apoptotic resistance to cisplatin exposure.

## Results

### N-Myc overexpression increased mitochondrial biogenesis

**Figure 1:**
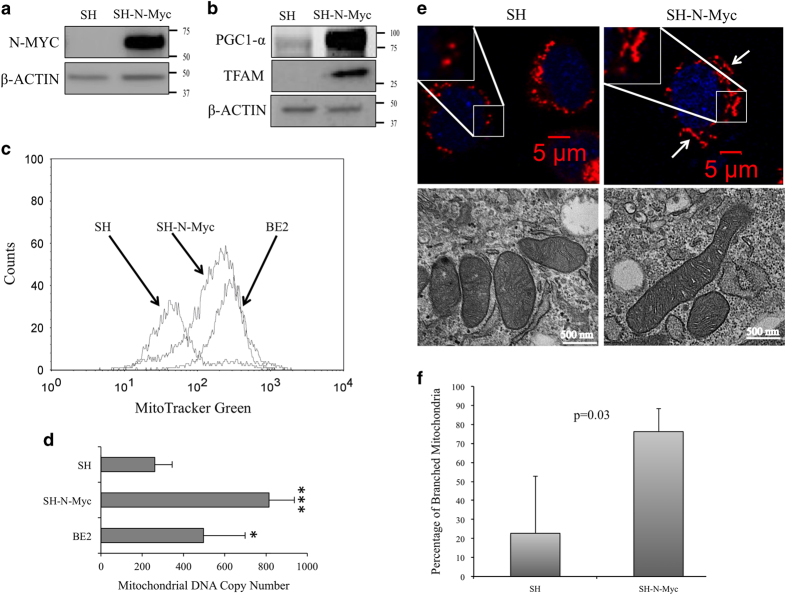
N-Myc overexpression increased mitochondrial biogenesis. (**a**) Whole cell lysates (WCL) from SH and SH-N-Myc cells were collected and used for western analysis with N-Myc antibodies that showed N-Myc was highly overexpressed in our model. (**b**) WCL were used to measure expression of the global mitochondrial regulators PGC1-a and TFAM. Both are upregulated in SH-N-Myc. (**c**) Cells at mid-logarithmic phase were stained with MitoTracker Green and measured by flow cytometry. A representative curve is shown. (**d**) A qPCR-based assay was used to measure mitochondrial DNA copy number using genomic DNA content as the control. Four separate experiments were performed with each cell line being measured at least in triplicate each time. Error bars show standard error of the experiments. *P* values: *P<0.05, ***P<0.0005. (**e**) The top row shows cells stained with MitoTracker Deep Red and DAPI followed by fixation. The insets zoom in on a part of the image that demonstrates the punctate nature of SH mitochondria and the elongated and branched structures in SH-N-Myc cells. The lower panel shows representative transmission electron images. (**f**) The percentage of branched mitochondria was calculated. Cells from above were randomly selected for manual counting of images. A minimum of 20 cells from three separate experiments were counted. Error bars show standard error of the experiments

As c-Myc overexpression increased mitochondrial biogenesis,^23,24^ we hypothesized that cultured human neuroblastoma cells would behave in a similar manner in response to N-Myc overexpression. SK-N-SH (SH) is a well established non-N-Myc amplified neuroblastoma cell line^30,31^ in which we ectopically overexpressed wild-type full-length human N-Myc (SH-N-Myc). This resulted in a 21-fold increase in N-Myc protein expression when compared with SH cells transfected with an empty vector (Figure 1a; relative expression: SH=1±0.08, SH-N-Myc=20.8±6.0).

Proper assessment of mitochondrial biogenesis requires a multi-faceted approach given its inherent complexity.^32^ PGC1-*α* is a master regulator of nuclear-encoded mitochondrial genes, and its expression was increased in SH-N-Myc cells (Figure 1b; relative expression: SH=1±0.02, SH-N-Myc=6.7±0.5). Furthermore, TFAM, a protein downstream of PGC1-*α* that regulates the transcription of the mitochondrial genome, was virtually undetectable in SH cells, yet was expressed in SH-N-Myc cells (Figure 1b).

Mitochondrial mass was measured by staining cells with a mitochondrial specific dye. SH-N-Myc cells showed an increase in fluorescence compared to SH cells (Figure 1c). This compared favorably with what we observed in BE2 cells, which is an established neuroblastoma cell line with N-Myc amplification (Supplementary Figure 1). We estimated the increase in mass in response to N-Myc overexpression to be about fourfold (Supplementary Figure 2).

We used real-time PCR to calculate mitochondrial DNA copy-number utilizing genomic DNA content as the control. SH-N-Myc cells had 2.5-fold more copies of the mitochondrial genome than controls (Figure 1d; SH=308±1.5, SH-N-Myc=770±412.2). BE2 cells also showed an increase in mitochondrial DNA (496.3±202.1) when compared with SH cells.

Using confocal microscopy, we noted the mitochondria of SH-N-Myc cells to be more tubular, elongated and branched than those in SH cells (Figure 1e, top). Consistent with this qualitative evaluation, the percentage of branched mitochondria was estimated to be threefold greater in SH-N-Myc cells (Figure 1f). This N-Myc-dependent elongation of the mitochondria was further evident by transmission electron microscopy (Figure 1e, bottom). The mitochondria of SH cells were mostly uniform in length, measuring ~1 *μ*m along the longest axis. In contrast, the length of the mitochondria of SH-N-Myc cells varied greatly, ranging from 0.5 to 2 microns. Collectively, these data demonstrated that overexpression of N-Myc increased mitochondrial biogenesis in neuroblastoma and that the organelle appeared more elongated and branched than those seen in control cells; therefore, we hypothesized that N-Myc overexpression increased mitochondria fusion.

### N-Myc overexpression deregulated mitochondrial dynamics

**Figure 2:**
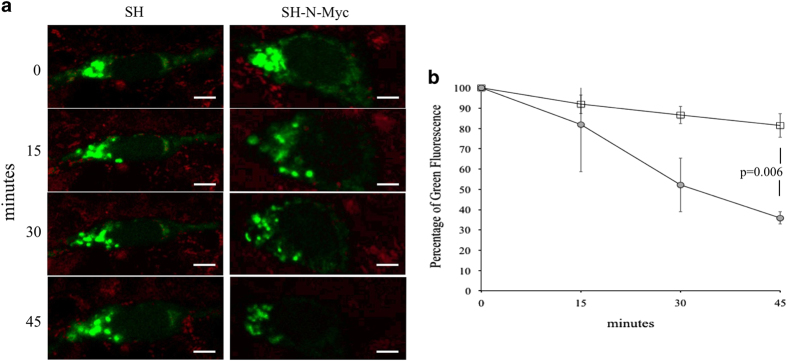
N-Myc overexpression deregulated mitochondrial dynamics. (**a**) SH and SH-N-Myc cells were transfected with a mito-PAGFP construct and were used for a mitochondrial fusion assay. Note the decay in the fluorescent signal in SH-N-Myc, which was not appreciated in SH cells. Cells were stained with MitoTracker Deep Red simultaneously to control for natural fluorescent decay. Reference bars represent 5 *μ*m. (**b**) Quantification of the green fluorescent signal over 30 individual cells subjected to the mitochondrial fusion assay. Error bars show standard error of the experiments

Mitochondrial dynamics are composed of two related, but opposing, pathways—fission and fusion. Mitochondrial fusion requires three mitochondrial membrane proteins: Mfn1; Mfn2; and Opa1. To quantify mitochondrial fusion, cells were transfected with a mitochondrially targeted photoactivatable GFP (Figure 2a). A subset of the mitochondrial biomass was stimulated by light, which activated the fluorescence only in that area. As the non-activated mitochondria fused with the fluorescent ones, the total signal decreased over time; the rate at which this occurred was directly proportional to the rate of fusion (Figure 2a). Over 45 min, SH cells retained 81.5±5.9% of the total green fluorescence, whereas SH-N-Myc only retained 35.9±3.0% (Figure 2b). To ensure that this decrease in signal was not secondary to mitochondrial loss, both cell lines were simultaneously stained with a red mitochondrial dye, which showed no difference in its decay (Supplementary Figure 3). From this we concluded that overexpression of N-Myc in neuroblastoma increased mitochondrial fusion by twofold.

**Figure 3:**
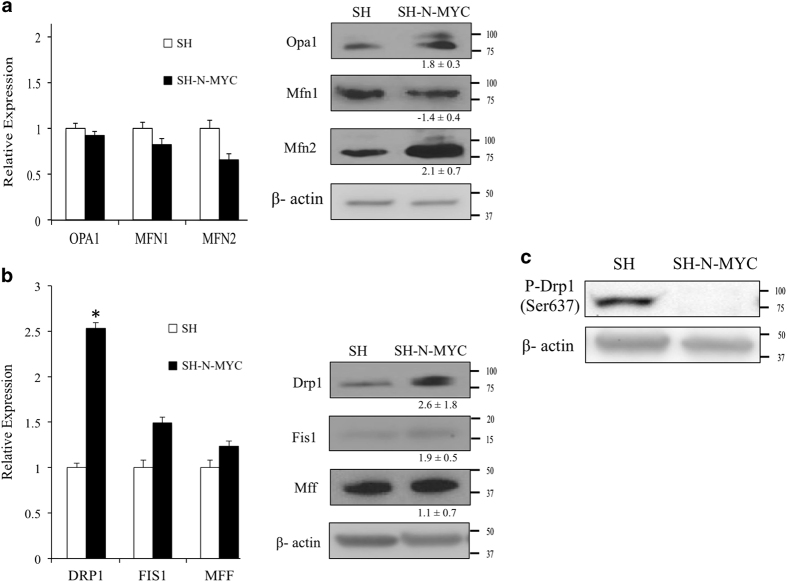
N-Myc overexpression deregulated mitochondrial dynamics. (**a**) The left panel shows logarithmically growing cells that were interrogated by qRT-PCR for the fusion genes. Experiments were performed in quadruplicate with a minimum of four repeats in each experiment. Right panels show westerns blots of WCL from the same cells. Densitometry was used to quantify the expression of the westerns of a minimum of four separate experiments. Data are expressed as relative fold change in expression in SH-N-Myc cells, with the expression in SH=1. Asterisk denotes *P*>0.05. (**b**) Fusion genes and their products were measured in the same manner as described above. (**c**) Western blots of WCL were used to detect Ser^637^-phopho-Drp1

An increase in mitochondrial fusion could be direct (via an increase in fusion) or indirect (a decrease in fission) or both. Given the fact that N-Myc is a global transcription factor, we sought to determine whether N-Myc could regulate the expression of the genes involved in these processes. We performed qRT-PCR and Westerns for the best described gene products of both mitochondrial fusion and fission. We found that N-Myc did not impose significant transcriptional control on the genes involved in fusion (Figure 3a, left). In fact, the only change that approached statistical significance was a 35% reduction in the *MFN2* transcript. Although there was minimal regulation at the transcriptional level, the expression of both Opa1 and Mfn2 proteins approximately doubled in SH-N-Myc cells (Figure 3a, right).

There are a number of proteins that have been implicated in mitochondrial fission. The process begins with the translocation of Drp1 from the cytosol to the outer mitochondrial membrane where it interacts with various proteins, including Mff and Fis1, prior to cleavage plane formation.^33^ Surprisingly, the most significant change in expression in SH-N-Myc cells was a 2.5-fold increase in the *DRP1* transcript (Figure 3b, left). The *MFF* and *FIS1* transcripts were also increased, albeit by >50% and did not rise to the level of significance. The Drp1 protein was increased 2.5-fold in SH-N-Myc cells (Figure 3b, right), which was consistent with transcript levels. Fis1 also increased twofold, however, Mff showed no significant change in protein expression.

The upregulation of Drp1 in SH-N-Myc cells was unexpected given the increased mitochondrial fusion observed in these cells; therefore, we examined the phosphorylation status of Drp1 as a possible explanation for this contradiction. We detected differential phosphorylation at Ser^637^; there was significantly less phopho-Drp1 detected in SH-N-Myc cells when compared to SH cells (Figure 3c). This modification has been shown to both inhibit^34^ and enhance^35^ the ability of Drp1 to initiate fission dependent on the kinase and the cellular context;^36^ therefore, it is possible that this change may be functionally relevant.

### Mitochondrial membrane potential increased with N-Myc overexpression

**Figure 4:**
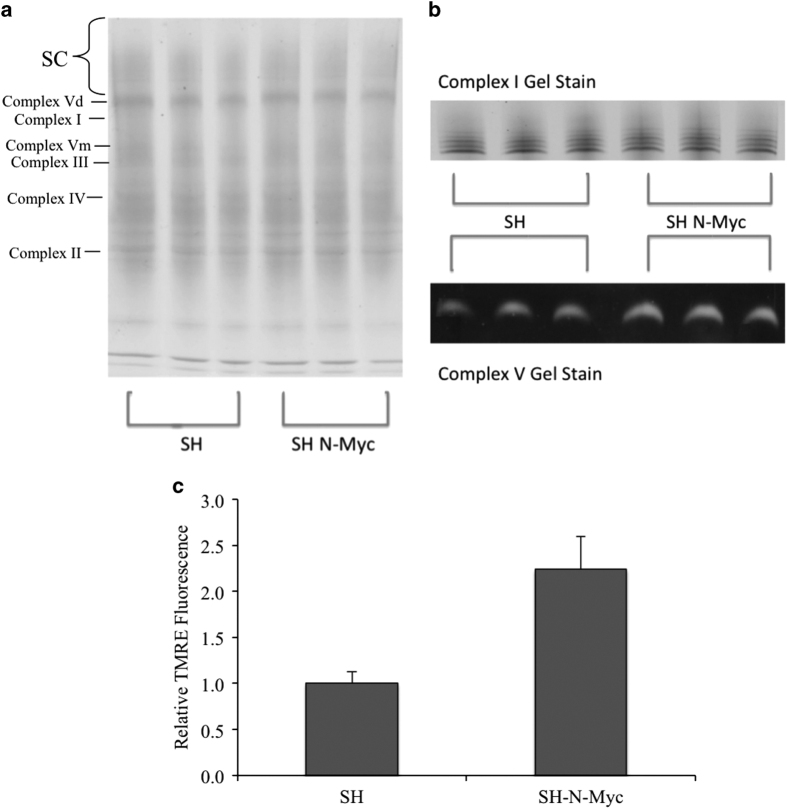
Mitochondrial membrane potential increased with N-Myc overexpression. (**a**) Blue native gel electrophoresis showed no discernable difference between ETC complexes from isolate mitochondria from the cells. (**b**) *In situ* enzymatic activities of Complexes I and V showed no apparent difference between the two cell lines. (**c**) Flow cytometry of cells stained with TMRE showed an increase in MMP in SH-N-Myc cells

There are many potential advantages for a cell with a more fused mitochondrial biomass,^37^ including enhanced diffusion of components of the electron transport chain (ETC).^38,39^ As N-Myc overexpression increased fusion in neuroblastoma cells, we sought to determine whether ETC complex formation was affected. There were no appreciable differences in the levels of any of the observed ETC complexes, including no alterations in supercomplex formation (Figure 4a). Furthermore, *in silica* enzymatic analysis showed no difference in the activity of either Complexes I or V (Figure 4b). From these data we concluded that the increased mitochondria in SH-N-Myc cells are replete with a normal and functional ETC.

To assay mitochondrial membrane potential (MMP) we stained cells with tetramethylrhodamine ethyl ester (TMRE) and found a 2.2-fold increase in fluorescence in SH-N-Myc cells, which is consistent with increased MMP (Figure 4c). We also used the mitochondrial specific dye, JC-1 (Supplementary Figure 4). Confocal microscopy showed that the punctate mitochondria of SH cells appeared mostly green, which indicates a reduced MMP, whereas the mitochondria of SH-N-Myc cells were primarily red and yellow indicating that the overall membrane potential was higher in these cells. Of note, the green mitochondria within the SH-N-Myc cells appeared more punctate, suggesting that the longer, more tubular mitochondria displayed a higher MMP. In summary, the mitochondria in the SH-N-Myc cells had no obvious structural or functional alterations in the ETC and therefore should be capable of undergoing OXPHOS efficiently. Given these findings, we hypothesized that ATP levels would be elevated in SH-N-Myc cells.

### N-Myc overexpressing cells rely on glycolysis for ATP production

**Figure 5:**
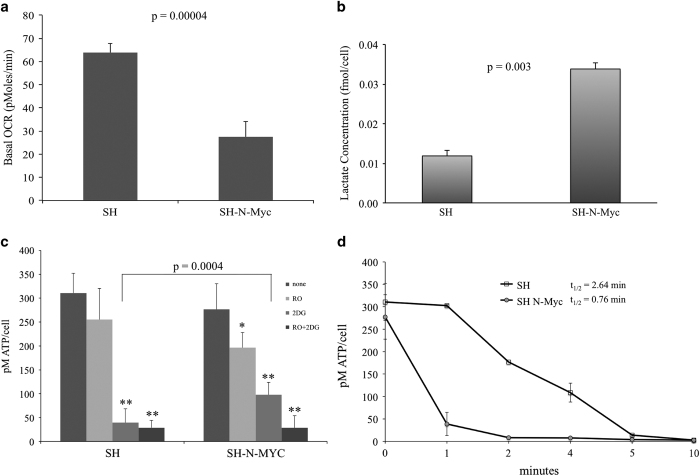
N-Myc overexpressing cells rely on glycolysis for ATP production. (**a**) Basal OCR was measured with the Seahorse Flux Analyzer. Five repeats were used for each cell line. (**b**) Lactate excretion was measured after 24 h in culture. Experiment was performed in quadruplicate. (**c**) Net ATP levels appeared the same in both cell lines growing under normal conditions. SH-N-Myc appeared to be less sensitive to 2DG suggesting the ability to better utilize OXPHOS when compared to SH cells. *P*-values: *P<0.005, **P<0.0005. (**d**) ATP half-life was assessed by measuring ATP levels following the addition of OXPHOS and glycolytic inhibitors together at time 0. In the presence of N-Myc amplification, there is a significant and sudden decrease in ATP levels in the presence of inhibitors. When inhibitors were added to SH cells, the decrease in levels was more gradual. ATP levels were nearly undetectable in SH-N-Myc cells by two minutes following complete metabolic inhibition. *P*-values for the 1, 2 and 4 min time points were <0.0005

We measured basal OXPHOS levels using a flux analyzer and found that the oxygen consumption rate was 2.3-fold higher in SH cells in comparison with SH-N-Myc cells (Figure 5a). These data were consistent with our measurement of oxygen consumption using a respirometer, which despite assaying the same amount of cellular lysate, showed no significant difference in oxygen consumption (Supplementary Figure 5). As we had established that SH-N-Myc cells had a larger mitochondrial mass (Figure 1), we can conclude that OXPHOS was actually lower in comparison with SH cells. Concomitantly, SH-N-Myc overexpressing cells excreted 2.8-fold more lactate than controls (SH=0.012±0.001 fmol/cell, SH-N-Myc=0.034±0.003 fmol/cell; Figure 5b) under standard growth conditions; therefore, we can conclude that N-Myc overexpression upregulated glycolysis.

There was no significant difference in ATP levels between the two cell lines (SH=310.8±41.4, SH-N-Myc=276.5±53.5 pM per cell). To inhibit OXPHOS, cells were incubated with oligomycin and rotenone, which blocked the activity of Complex V and I of the ETC, respectively; this blockade required that ATP be generated via glycolysis. There was a small decrease in ATP levels when both cell lines were exposed to these inhibitors (Figure 5c). To better determine the contribution of glycolysis to ATP production, cells were incubated with 2-deoxy-D-glucose (2DG), which decrease ATP levels in SH cells by 80% and by 60% in SH-N-Myc cells. The addition of all inhibitors resulted in a 90% decrease in ATP levels in both cells types. Collectively, these data suggested that both SH and SH-N-Myc cells relied mostly on glycolysis for ATP production.

As cells that overexpress Myc proteins are known to have increased metabolic demands, we found it surprising that ATP levels were not comparatively increased in SH-N-Myc cells. As the previous experiments only measured net ATP levels, we sought to separate production from consumption by inhibiting cells with 2DG, rotenone and oligomycin simultaneously, which should stop all ATP synthesis. We found that ATP rapidly decreased in SH-N-Myc cells in comparison to controls (Figure 5d; *t*_1/2_: SH=2.64 min, SH-N-Myc=0.76 min), indicating increased ATP consumption. This also implied that in order to maintain the same net ATP levels as SH cells, SH-N-Myc cells had increased ATP production. From this we can conclude that SH-N-Myc cells had both increased ATP turnover and synthesis, secondary to an upregulation of glycolysis, despite the increased mitochondrial biomass.

### N-Myc overexpressing cells protect against cisplatin-induced apoptosis

**Figure 6:**
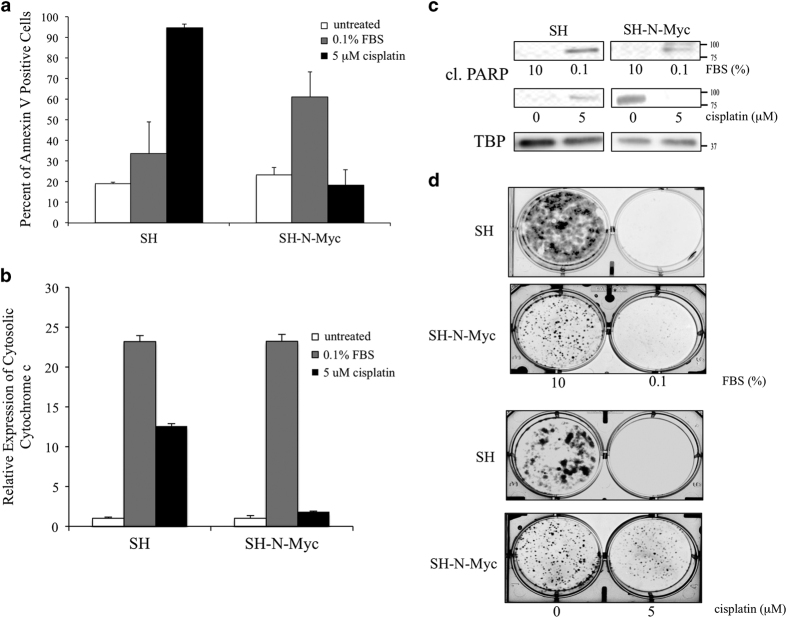
N-Myc overexpressing neuroblastoma cells are resistant to apoptosis in response to cisplatin exposure. (**a**) Representative flow cytometry diagrams of cells exposed to DMEM with: 10% (untreated), 0.1% fetal calf serum (FCS), or 5 *μ*M cisplatin for 48 h, and then cells were stained with Annexin V. (**b**) Quantification of densitometry of the expression of cytochrome *c* detected in the cytoplasmic fraction of cells detected by western blotting. Values were made relative to untreated samples of each cell type. (**c**) Cells were fractionated after 48 h of growth in the respective media and nuclear fractions were analyzed by western blots to detect cleavage of PARP. TBP was used as the loading control. (**d**) Clonogenic plate assays were performed after 5 days of growth in the respective media

As increased mitochondrial fusion has been implicated in increasing apoptotic resistance,^40–42^ we hypothesized that SH-N-Myc cells would be less able to undergo programmed cell death than SH cells. We used two types of apoptotic induction—serum withdrawal and exposure to cisplatin. Reducing the concentration of fetal bovine serum (FBS) in the growth medium is known to stimulate apoptosis in neuroblastoma cells^43,44^ and has also been shown to induce an exaggerated response in Myc overexpressing cells.^45^ Peak levels of apoptosis were achieved after 48 h of exposure to media containing 0.1% FBS; the SH-N-Myc cells showed ~50% apoptosis as measured by Annexin V positivity, which was a twofold increase when compared with SH cells (Figure 6a). This correlated with an increase of cytochrome *c* in the cytosol (Figure 6b) and increased detection of cleaved PARP (Figure 6c). Clonogenic colonies assays were performed as a surrogate measure of tumorgenicity and response to apoptotic stimuli. Both cell lines grown in 0.1% FBS for five days showed a marked decrease in colony formation (Figure 6d). From this we conclude that both SH and SH-N-Myc cell lines are competent to undergo apoptosis.

Cisplatin, which is a mainstay of neuroblastoma therapy, causes DNA damage that subsequently induces apoptosis. We exposed our cells to media containing 5 *μ*M cisplatin and observed that SH cells were 80% apoptotic by 48 h, whereas SH-N-Myc cells showed no appreciable Annexin V positivity (Figure 6a). SH cells exposed to cisplatin also showed increased cytosolic cytochrome *c* (Figure 6b), and cleavage of PARP (Figure 6c), whereas SH-N-Myc cells did not (Figures 6b and c). Consistently, 5 *μ*M cisplatin completely eliminated colony formation in SH cells, whereas SH-N-Myc cells were able to form colonies at levels identical to the unexposed cells (Figure 6d). These data demonstrated that N-Myc overexpression increased cisplatin resistance in SH cells.

**Figure 7:**
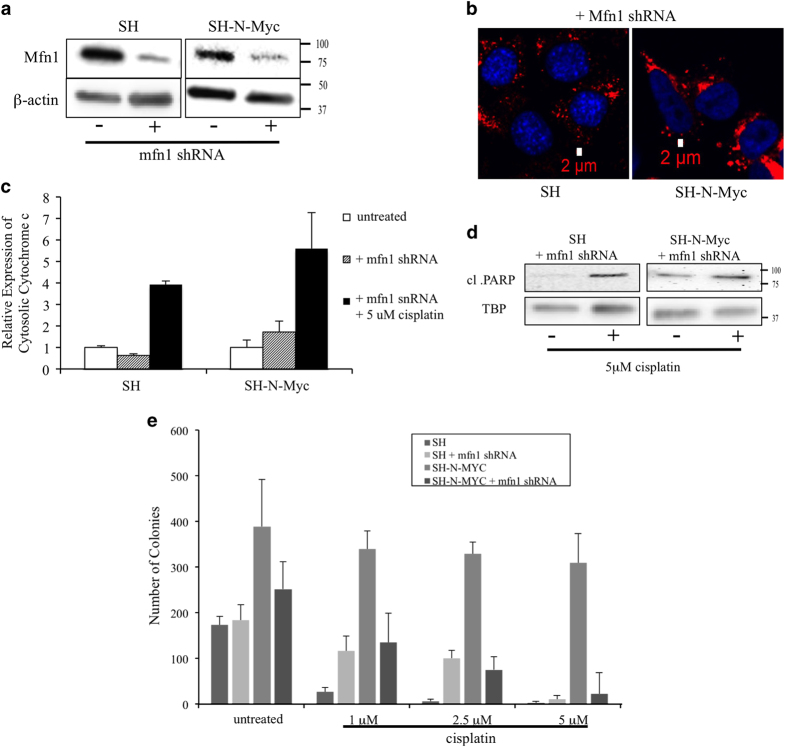
Reduction of MFN1 expression restores apoptotic sensitivity to cisplatin. (**a**) Cells were transfected with a Mfn1-specfic shRNA and compared with cells transfected with a scrambled shRNA. Mfn1 expression was reduced in both cell lines. (**b**) Fluorescent microscopy of cells stained with MitoTracker Deep Red and DAPI show that the mitochondria in SH-N-Myc cells have become more punctate following transfection with Mfn1 shRNA compared with non transfected controls (Figure 1e). (**c**) Quantification of densitometry of the expression of cytochrome *c* detected in the cytoplasmic fraction of cells detected by western blotting. Values were made relative to untreated samples of each cell type. Note that reduction of Mfn1 in SH-N-Myc cells now increases the appearance of cytosolic cytochrome *c*. (**d**) Western blots showed the cleavage of PARP. Note the appearance of cleaved PARP in SH-N-Myc+Mfn1 shRNA cells exposed to cisplatin. (e) Quantification of clonogenic plate assays repeated a minimum of six times. Cells were exposed to a range of cisplatin concentrations from 0–10 *μ*M

Previous studies suggested that increased mitochondrial fusion increased cisplatin resistance;^41^ therefore, we hypothesized that the increased fusion seen in SH-N-Myc cells may be the mechanism underlying this finding. To challenge this, we transfected SH±N-Myc cells with an shRNA targeting the *MFN1* gene. Mfn1 is a protein involved in mitochondrial fusion and decreased levels have been shown to result in more punctate mitochondria.^46^ We reduced Mfn1 protein levels by two- to fourfold in both cell lines (Figure 7a), which resulted in a more punctate appearance (Figure 7b), and a decrease in branching of the mitochondria (Supplementary Figure 6). Following exposure to 5 *μ*M cisplatin for 48 h, cytosolic cytochrome *c* (Figure 7c) and cleaved PARP (Figure 7d) were detected in both SH as well as SH-N-Myc cells transfected with mfn1 shRNA. This showed that reduction of Mfn1 sensitized SH-N-Myc cells to apoptotic induction by cisplatin. In addition, we performed clonogenic plate assays and found decreased colony formation in SH-N-Myc+ mfn1 shRNA cells growing in the presence of cisplatin. Cells grown in only 1 *μ*M cisplatin showed a threefold reduction in colony formation and was completed eliminated in 5 *μ*M cisplatin (Figure 7e). Surprisingly, SH+ mfn1 shRNA cells displayed more colonies than SH cells at cisplatin concentrations less than 5 *μ*M. Finally, SH-N-Myc+ mfn1 shRNA cells plated in the absence of cisplatin showed a 30% reduction in colony formation compared with control cells, suggesting that reducing Mfn1 has deleterious effects exclusively on SH-N-Myc cells. From these data we concluded that Mfn1 expression, and subsequently mitochondrial fusion, is likely to be required for the apoptotic resistance to cisplatin exposure seen in SH-N-Myc cells.

## Discussion

Amplification of N-Myc is the most significant negative prognostic factor in neuroblastoma,^1^ yet there have been limited studies addressing the resulting phenotypic changes. This study focuses on the effects of N-Myc amplification on mitochondrial biogenesis.

Mitochondrial biogenesis is a multistep process that includes: mitochondrial protein synthesis and modifications^47,48^ and conservation and reorganization of the mitochondrial biomass by fusion and fission.^49–51^ Increased mitochondrial biogenesis may provide a transformed cell with a growth advantage by increasing its OXPHOS capacity in order to produce the ATP required for increased growth and proliferation and/or providing the necessary machinery to meet the increased demands of amino acid and nucleotide synthesis at the expense of OXPHOS.^24,52,53^

We utilized cultured neuroblastoma cells with normal endogenous N-Myc expression (SH) as the control in which N-Myc was ectopically overexpressed constitutively (SH-N-Myc) to determine the impact of N-Myc because they were isogenic and c-Myc was undetectable (Supplementary Figure 7), which eliminated this as a confounding factor. Initially our study also examined neuroblastoma cells with naturally amplified N-Myc (BE2). As the findings in SH-N-Myc and BE2 were identical, we chose to focus on the isogenic cells in order to better establish causality.

SH-N-Myc cells had increased mitochondrial biogenesis when compared with SH cells by a number of quantifiable measures: a 4-fold increase in mitochondrial biomass, a 2.5-fold increase in mitochondrial DNA copy number and increased elongation and branching of the mitochondria (Figure 1). We believe this represents the first example of overexpression of N-Myc deregulating mitochondrial biogenesis. One of the most defining characteristics of mitochondria from N-Myc overexpressing neuroblastoma cells was the appearance of supraphysiologically elongated mitochondria; we hypothesized this to be a result of dysregulated mitochondrial dynamics. Consistent with this, we found mitochondrial fusion was twofold higher in SH-N-Myc cells. Interestingly, most of the major proteins involved in both fusion and fission were increased by 50–100% in SH-N-Myc cells. The fact that proteins of opposing functions showed increased expression, yet the sum result was an increase in mitochondrial fusion, suggested that modifications of the proteins may be important in their regulatory control, consistent with previous studies.^36,54,55^ We were surprised that the most overexpressed protein in SH-N-Myc cells was Drp1, which led us to interrogate the phosphorylation status of the protein, as this is the primary method by which its function is regulated. The phenotypic effect of phosphorylation is highly context dependent;^34,36,56^ for example, phosphorylation by either CaMKI*α*^57^ or ROCK1^35^ has been shown to stimulate mitochondrial fission by increasing the affinity of Drp1 for Fis1, which is localized to the mitochondrial membrane.^36^ We are currently exploring how modification of Drp1 contributes to mitochondrial structure and function in response to N-Myc overexpression. Interestingly, the most significant change in transcript expression was *DRP1*. We speculate that the increased expression in SH-N-Myc cells was compensatory; we believe that *DRP1* was transcriptionally upregulated by N-Myc, in an effort to maintain mitochondrial dynamic homeostasis. Currently, we are determining whether *DRP1* is a direct N-Myc target and the context of this regulation.

Mitochondrial fusion may be advantageous for tumor growth^58,59^ and inhibiting apoptosis.^28,41^ Cells with increased mitochondrial fusion are more efficient at ATP production,^60^ which could aid in tumorigenesis; therefore we predicted that SH-N-Myc cells have increased OXPHOS secondary to increased fusion and biomass. Unexpectedly, SH-N-Myc showed reduced oxygen consumption. This was not explained by structural abnormalities, as SH-N-Myc cells displayed normal stoichiometry of the ETC complexes (Figure 4a) and a twofold increase in MMP (Figure 4c). Despite a decrease in OXPHOS, SH-N-Myc cells had the same net ATP levels as SH cells (Figure 5c); however, this occurred after ATP was utilized more rapidly in SH-N-Myc cells. This suggested that ATP production was increased (Figure 5d). We showed that glycolysis was upregulated in SH-N-Myc cells as lactate excretion was elevated. We believe this increase in glycolysis produced the ATP necessary to maintain homeostasis.

Mitochondrial dynamics proteins interact with the apoptotic pathways in a complex manner. One consequence of these interactions is that mitochondrial fission is a prerequisite step to the intrinsic apoptotic pathway. It is believed that mitochondrial fusion reduces apoptosis by its natural opposition to fission. In our study N-Myc overexpression increased apoptosis in response to serum withdrawal, following release of cytochrome c into the cytosol and subsequent cleavage of proteins such as PARP (Figure 6).

Cisplatin is a mainstay in neuroblastoma treatment and functions through the generation of DNA adducts, which induce apoptotic pathways. In our study concentrations as low as 1 *μ*M induced apoptosis in SH cells within 24 h. In contrast, SH-N-Myc cells were resistant to apoptosis when exposed to 5 *μ*M cisplatin, and relatively resistant to concentrations as high as 20 *μ*M (Supplementary Figure 8). Although other studies showed an elevated copy number of N-Myc induced apoptotic resistance,^61–64^ a study by Fulda *et al.*^65^ concluded that N-Myc sensitized cells to cisplatin. A detailed look revealed that the concentrations at which cells died *in vitro* was in excess of what would be the expected mean plasma levels of children receiving cisplatin,^66^ and cells overexpressing N-Myc were actually resistant to apoptosis at cisplatin concentrations closer to those used in our study.^65^ Interestingly, despite using the same SH cells utilized in our study as their low N-Myc expression control, those cells did not display apoptosis upon cisplatin exposure in their hands. As they used an inducible N-Myc expression construct, we speculate that their N-Myc expression was not tightly regulated.

A study found a positive correlation between levels of mitochondrial fusion and cisplatin resistance in neuroblastoma;^41^ therefore, we hypothesized that the ability of N-Myc to protect cells against apoptosis was via their ability to upregulate fusion. We reduced expression of Mfn1 to inhibit mitochondrial fusion, as it is believed to function exclusively in mitochondrial fusion.^67^ Decreased Mfn1 eliminated the protection from cisplatin exposure conferred by N-Myc overexpression. This appeared to be specific to SH-N-Myc cells, as reduction of Mfn1 in the SH cells actually reduced apoptosis at lower concentrations of cisplatin, which we hope to understand better in future studies, yet believe further strengthens our finding that the reversal of the apoptotic resistance is specific to cells overexpressing N-Myc. Furthermore, we speculate that SH-N-Myc cells become ‘addicted’ to the increased mitochondrial fusion for apoptotic resistance and may be responsible for some of the pathology of this tumor.

## Materials and methods

### Cell culture

The neuroblastoma cell line SK-N-SH (SH) was used, as it has been well-characterized and known to have normal diploid expression of *NMYC*.^68^ SH cells were transfected with a construct that contained the *NMYC* gene whose expression was driven by a constitutively active CMV promoter. Transfections were performed using the MegaTran transfection reagent (OriGene, Rockville, MD, USA) according to the manufacturer’s instructions. Stable pooled tranductants were selected by resistance to G418. All cell lines were grown in Dulbecco’s modified minimal essential media (DMEM) containing 10% FBS, glutamine and penicillin plus streptomycin as previously described.^69^ The SH-N-Myc cells were maintained in 10 *μ*g/ml G418 and the media was replaced every 48–72 h. All recombinant DNA work was approved by the University of Pittsburgh Institutional Biosafety Committee and was performed under BSL2^+^ conditions.

### Immunoblotting

Cells were grown to approximately 75–90% confluency under standard conditions and then harvested with trypsin. After washing in PBS, cell pellets were lysed in SDS-PAGE lysis buffer. Five microgram of total protein lysate from each cell line was then resolved on a 10% SDS-polyacrylamide gel and transferred to a PVDF membrane (Millipore, Inc., Bedford, NY, USA) by electrophoretic transfer according to manufacturer’s instructions (Bio-Rad). Immunoblotting was performed as previously described.^24^ The antibodies used in this study are as follows: anti-N-Myc (1 : 200, Santa Cruz Biotechnology, Inc., Dallas, TX, USA, #sc-53993), anti-Opa1 (1 : 5000, BD Biosciences, San Jose, CA, USA, #612606, Beverly, MA, USA), anti-Mfn1 (1 : 1000, Santa Cruz Biotechnology, Inc., #sc-100560), anti-Mfn2 (1 : 500, Santa Cruz Biotechnology, Inc., sc-100560), anti-Drp1 (1 : 5000, BD Biosciences, #611112), anti-Mff (1 : 500, Proteintech Group, #170901-1-AP, Chicago, IL, USA), anti-c-Myc (1 : 500, Santa Cruz Biotechnology, Inc., #sc-40), anti-VDAC (1 : 1000, Abcam, #AB14734, Cambridge, MA, USA), and anti-*β*-Actin (1 : 10 000, Cell Signaling Technology, Danvers, MA, USA #4967S). Secondary antibodies used were anti-mouse and anti-rabbit HRP-linked antibodies (1 : 10 000, Cell Signaling Technology; #7076S, #7074P2, respectively). Westerns were each repeated a minimum of three separate times with fresh lysates. Densitometry was performed using GelEval software (FrogDance Software, Dundee, UK).

### Quantitation of mitochondrial mass and membrane potential

Mitochondrial mass was measured by staining cells grown to 75% confluency in a six-well dish with 0.5 *μ*M MitoTracker Green(Invitrogen, Carlsbad, CA, USA) and 1 *μ*M 10-nonyl acridine orange (NAO) as previously described.^24^ To measure MMP, cells were stained with 0.1 *μ*M TMRE, which is a positively charged fluorescent molecule that accumulates and is retained in the negatively charged polarized mitochondrial matrix. Cells were washed with PBS, collected by scraping and analyzed using a FACStar flow cytometer (BD Biosciences) to fluoresce the respective dyes. To observe by confocal microscopy, cells were stained directly in glass bottom 60 mm tissue culture dishes, washed with PBS and examined as described above.

In addition, cells were plated on glass bottom 60 mm tissue culture dishes at 50% confluency and stained with 0.5 *μ*M JC-1 dye (Invitogen) for 25 min at 37 °C and 5% CO_2_. Cells were washed with PBS and confocal images were taken as described above. Relative degrees of mitochondrial polarization were quantified by measuring the ratio of red-shifted JC-1 aggregates, which are favored under conditions of high membrane potential, and green-shifted monomers, which tend to predominate under conditions of low membrane potential.^70^

### Mitochondrial DNA content

Logarithmically growing cells were collected and used for quantification of mitochondrial to nuclear DNA content according to the protocol provided for the Human Mitochondrial DNA (mtDNA) Monitoring Primer Set (Takara Bio, Inc., Mountain View, CA, USA). The experiment was performed in quadruplicate.

### Fluorescence microscopy

Cells were grown to 50% confluency on glass-bottom 60 mm tissue culture plates and stained with a mitochondrial specific dye as indicated below. Using a Zeiss LSM710 confocal microscope at 0.5 *μ*m intervals, high power (×60) fluorescent images were obtained while being maintained at 37 °C and 5% CO_2_. Fluorescence was quantified with the ImageJ software (Madison, WI, USA), using previously described methods.^71^

### Electron microscopy

Cells were grown in six-well tissue culture dishes to 80% confluency and subsequently fixed with 2.5% glutaraldehyde. Samples were prepared as previously described,^72^ and then photographed using a JEM 1210 transmission electron microscope (JEOL, Peabody, MA, USA) equipped with a CCD camera (Advanced Microscopy Techniques Corp., Danvers, MA, USA) at 80 kV.

### Mitochondrial fusion assay

Cells were plated directed onto glass-bottom culture plates and transiently transfected with construct that expressed a mitochondrially targeted photoactivated GFP (mito-PAGFP was a gift from Richard Youle (Addgene plasmid # 23348)). Although being maintained at 37 °C and 5% CO_2_, the Zeiss LSM710 confocal microscope was used for the experiment. A small area of the mitochondrial biomass was stimulated by 488 nm light, which triggered the activation of the fluorescence, yet left the other mitochondria in the cell with minimal signal. As the non-activated mitochondria fused with the activated ones, the total fluorescent signal decreased with time, and the rate at which this occurred was directly proportional to the rate of mitochondrial fusion (Figure 2). To ensure that the decrease in signal was a result of fusion events as opposed to mitochondrial loss, the cells were simultaneously stained with the mitochondrial specific dye, MitoTracker Deep Red, whose signal was measured over time. The experiment was performed in triplicate with each experiment measuring at least 10 isolated cells.

### Quantitative real-time PCR

RNAs were prepared according to the protocol provided with the RNeasy Mini Kit (QIAGEN, Hilden, Germany) and stored at −20 °C. RNA was used for RT-PCR according to the protocol provided with the QuantiTect SYBR Green RT-PCR Kit (QIAGEN). The sequences for the primer pairs were obtained from the Primer Bank (http://pga.mgh.harvard.edu/primerbank/) and commercially synthesized (IDT, Coralville, IA, USA). Specific primer sequences are listed in Table 1. Samples were run on the Roche Light Cycler according to manufacturer’s specifications. Amplification of a PCR product of the predicted size was verified by electrophoresis of an aliquot of the final reaction on a 4% NuSieve Agarose gel (Cambrex, East Rutherford, NJ, USA). Relative values were calculated according to the ΔΔCT method^73,74^ using *β*-microglobin as the housekeeping gene and setting the expression of each gene relative to the levels seen in SH cells.

### Measurements of basal OCR

All measurements were performed with a Seahorse Bioscience XF24 Extracellular Flux Analyzer (Billirica, MA USA). Cells were plated at 20 000 cells per well onto Seahorse 24-well plates 12–18 h prior to the assay. Immediately following the addition of fresh medium, O_2_ consumption rate (OCR) were quantified to obtain baseline levels of these processes. Experiments were performed by simultaneously measuring three to five replicates of each cell line.

### Respirometer

SH and SH-N-Myc cells were cultured on 150 mm tissue culture plates. Cells were trypsinized, counted, washed and resuspended at 5×10^6^ in MiR06 respiration medium. Each chamber of the twin chamber Oroboros was filled with 2 ml of the cell suspension. 10 mg/ml of digitonin in DMSO was added to permeabilize the cells. The O_2_ flow signal was allowed to stabilize at a constant rate (routine respiration rate) before the addition of compounds for inhibiting electron transport chain (ETC) complexes. Glutamate, malate and pyruvate (GMP) substrates were added to prime Complex I before the addition of ADP. Succinate and rotenone were then added blocking Complex II and Complex I. Cytochrome C was added at the end to check for membrane integrity. The two samples were collected after completion to check protein levels.

Identical numbers of SH±N-Myc cells were introduced to the instrument followed by the sequential addition of several compounds. Initially, digoxin was added to permeable the cells, followed by simultaneous injection of glutamate, malate and pyruvate where provided substrate for proper Complex I activity. The full respiratory capacity was seen following the addition of ADP.

### Measurement of ATP levels

The ATPLite Luminescence Assay System (Perkin Elmer, Waltham, MA, USA) was used to measure ATP levels according to manufacturer's instructions. Twenty thousand cells were grown overnight in a 96-well plate and were exposed to 100 *μ*l of media with and without metabolic inhibitors at 37 °C for 45 min before running the assay. The inhibitors used were: oligomycin (1 *μ*M concentration) to inhibit Complex V, allowing protons to accumulate across the inner mitochondrial matrix; 2DG (100 mM concentration), to block glucose uptake thereby blocking glycolysis; rotenone (1 *μ*M concentration) to inhibit Complex I; and cycloheximide (50 *μ*g/ml concentration) to inhibit protein synthesis. Cells were incubated with cycloheximide for 2 h prior to the assay. The experiments were performed at least —two to four times with —three to six replicates for each experiment. Data were acquired on a 96-well plate reader that read luminescence.

To determine the half-life of ATP the cells were grown as described above, after which media containing 100 mM 2-DG, 10 *μ*M oligomycin and 10 *μ*M rotenone was added. On the basis of the manufacturer’s instructions, the provided cell lysis solution was added to stop the reaction at various time points (1–30 min). Luminescence was measured and the half-life was calculated based on an exponential line equation.

### Preparation of mitochondria

Approximately 1×10^8^ freshly collected SH and SH-N-Myc cells were suspended in 500 *μ*l of cold buffer 25 mM Tris-HCl, pH 7.5; 100 mM KCl; 0.4 M sucrose containing protease inhibitor cocktail (Sigma-Aldrich, St Louis, MO, USA) and disrupted in a cell homogenizer (Isobiotec, Heidelberg, Germany) for 50 strokes on ice. The resultant suspension was centrifuged at 4000×*g* for 15 min. The pellet was washed twice with PBS and centrifuged again at 1500×*g* for 15 min. The resultant pellet was suspended in the sucrose-containing PI cocktail.

### Blue native gel electrophoresis

Eight milligram of digitonin (MP Biomedicals, Solon, OH, USA) was dissolved in 200 *μ*l of 30 mM HEPES buffer, pH 7.4, 150 mM potassium acetate and 10% glycerol, heated at 95 °C, then cooled on ice. One milligram of mitochondria, isolated as described above, was lysed by the addition of digitonin to a final ratio of 1:8 g:g; protein : digitonin. Following incubation on ice for 20 min, a Coomassie blue solution (5% Coomassie blue G250 in 750 mM 6-aminocaproic acid) was added (1/20 v/v), and the mixture was centrifuged at 14 000×*g* for 20 min at 4 °C. The supernatant was then directly loaded onto a 3–12% Native PAGE Novex Bis-Tris gel (Invitrogen), and subjected to electrophoresis at 80 V for 4 h at 4 °C in the buffer provided by the supplier. Following electrophoresis, gels were stained with Bio-Safe Coomassie G250 (Bio-Rad, Hercules, CA, USA) for 30 min and exhaustively destained with water. Stained gels were scanned and the images analyzed for relative band density using AlphaEaseFC 2200 scanner and AlphaEaseFC software (San Leandro, CA, USA).

### Complexes I and V gel *in situ* activity stain

For Complex I activity measurements, the blue native gel was placed in 3–4 ml of 2 mM Tris-HCl, pH 7.4 buffer containing 2.5 mg/ml nitrotertrazolinum blue chloride and freshly added 0.1 mg/ml NADH. The gel was incubated at 37 °C for 1–2 h and then subjected to densitometric analysis as described above. An average value from three gels was calculated. To quantify the ATPase activity of Complex V, gels were incubated in 3–4 ml of 34 mM Tris-glycine, pH 7.8, 14 mM MgSO_4_, 0.2% Pb(NO_3_)_2_ with freshly added 8 mM ATP for 3 h at 37 °C. Bands were quantified as for Complex I.

### Statistical methods

*P* values were calculated by using the student *t*-test feature in Microsoft Excel.

## Figures and Tables

**Table 1:** Primers used in qRT-PCR

*Loci*	*FORWARD*	*REVERSE*
OPA1	5′- TGTGAGGTCTGCCAGTCTTTA-3′	5′- TGTCCTTAATTGGGGTCGTTG-3′
MFN1	5′- TGGCTAAGAAGGCGATTACTGC	5′- TCTCCGAGATAGCACCTCACC-3′
MFN2	5′- CTCTCGATGCAACTCTATCGTC-3′	5′- TCCTGTACGTGTCTTCAAGGAA-3′
DRP1	5′- AATCCTAATTCCATTATCCTCGCT-3′	5′- ACCAGTAGCATTTCTAATGGC-3′
FIS1	5′- GTCCAAGAGCACGCAGTTTG-3′	5′- ATGCCTTTACGGATGTCATCATT-3′
MFF	5′- ACTGAAGGCATTAGTCAGCGA-3′	5′- TTGGCGGTGCTACTTTTAACTT-3′
B2M	5′- ATTCACCCCCACTGAGACTGA-3′	5′- CTCGATCCCAGTAGACGGTC-3′

## Abbreviations

2DG: 2-deoxy-D-glucose
DMEM: Dulbecco's-modified minimal essential media
ETC: electron transport chain
FBS: fetal bovine serum
GMP: glutamate/malate/pyruvate
MMP: mitochondrial membrane potential
N-Myc: *N-MYC* gene product
OCR: oxygen consumption rate
OXPHOS: oxidative phosphorylation
SH: SK-N-SH
TMRE: tetramethylrhodamine ethyl ester.
CPC: Chromosomal Passenger Complex
DDR: DNA Damage Response
SAC: Spindle Assembly Checkpoint.
