# Supplementary material for: N-Myc overexpression increases cisplatin resistance in neuroblastoma via deregulation of mitochondrial dynamics
Source: Cell Death Discov. 2016 Dec 12;2:16082–. doi: 10.1038/cddiscovery.2016.82 (PMC5149579; doi:10.1038/cddiscovery.2016.82)
Supplement: Supplementary Figures [file cddiscovery201682-s1.pdf]

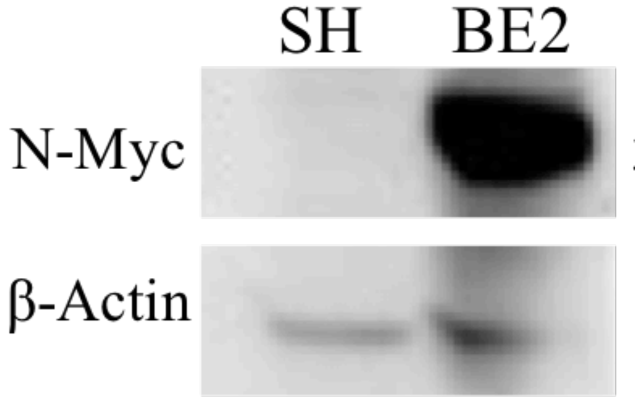

Supplemental Figure 1

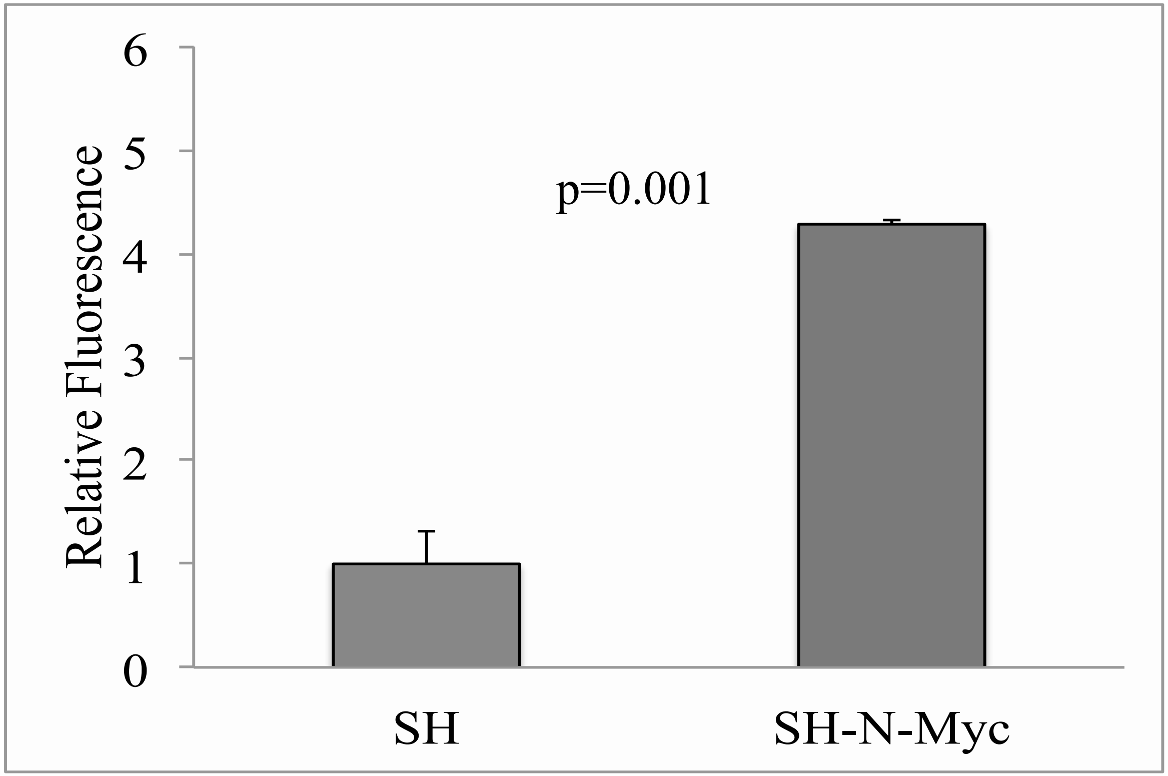

Supplemental Figure 2

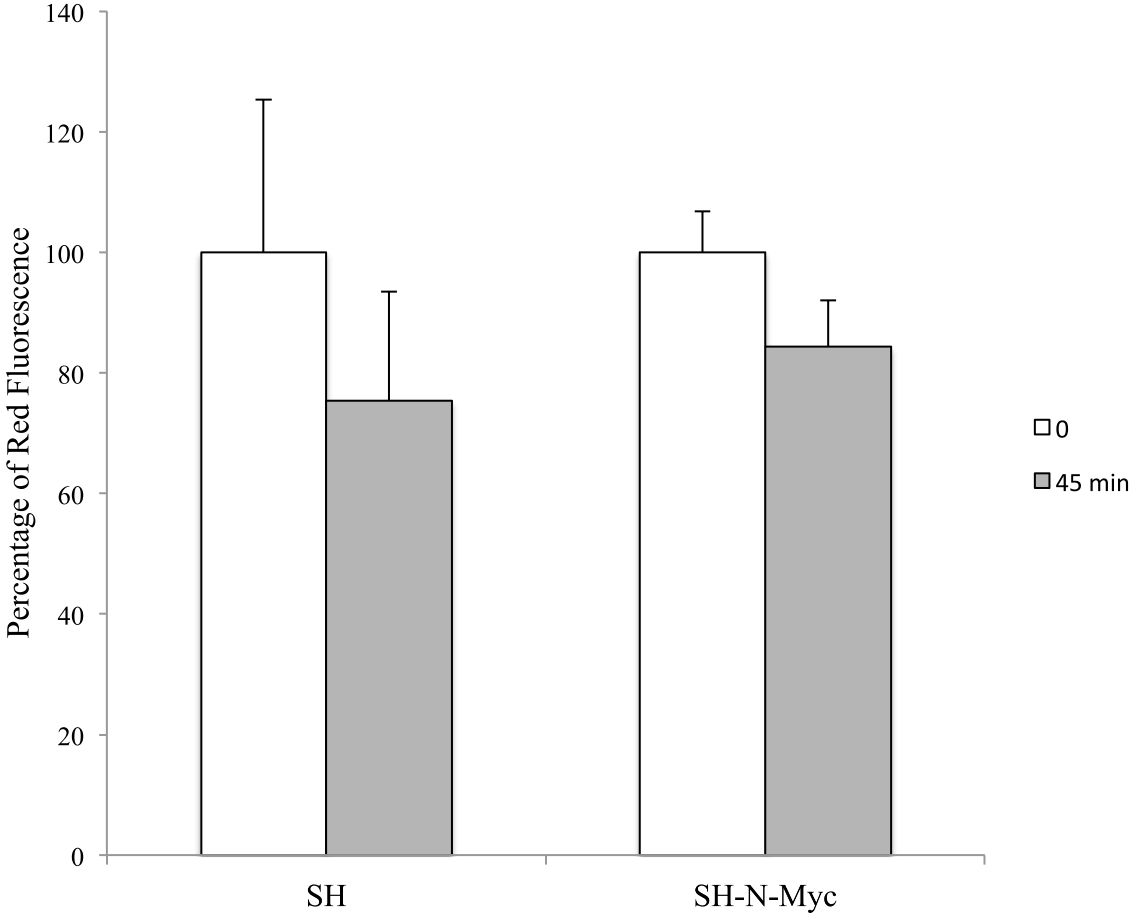

Supplemental Figure 3

p = 0.007

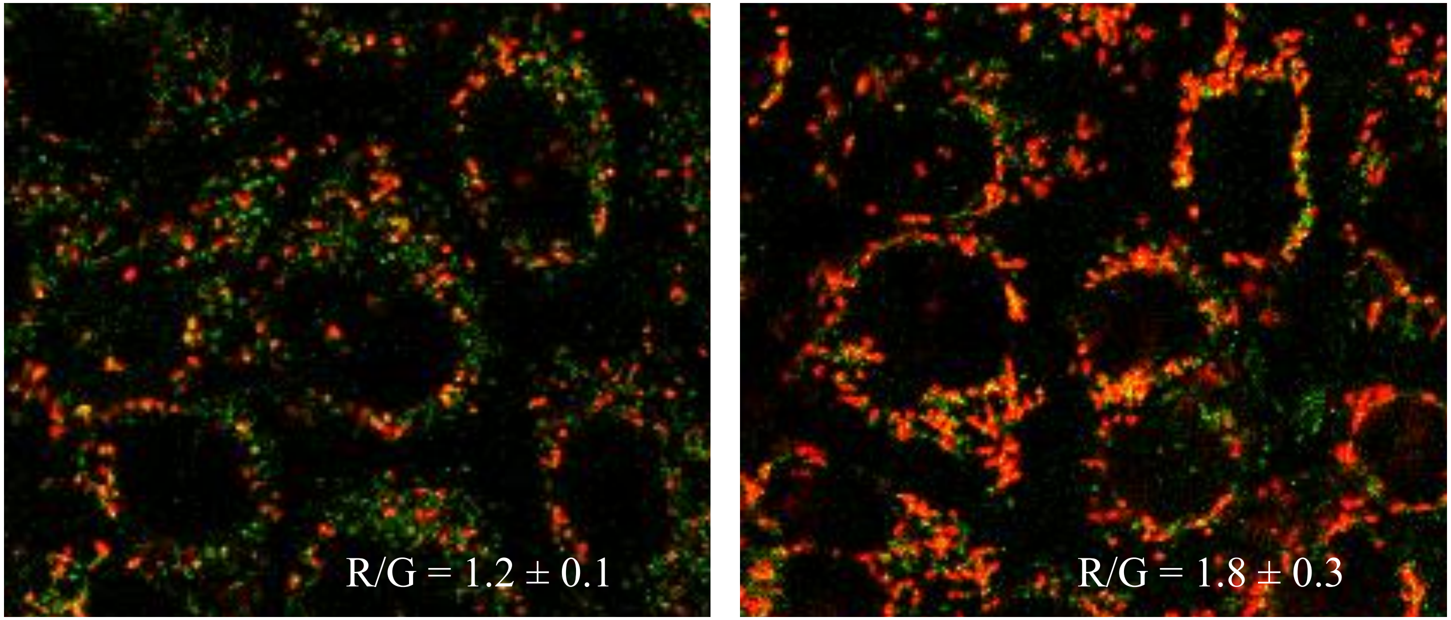

SH

SH-N-Myc

Supplemental Figure 4

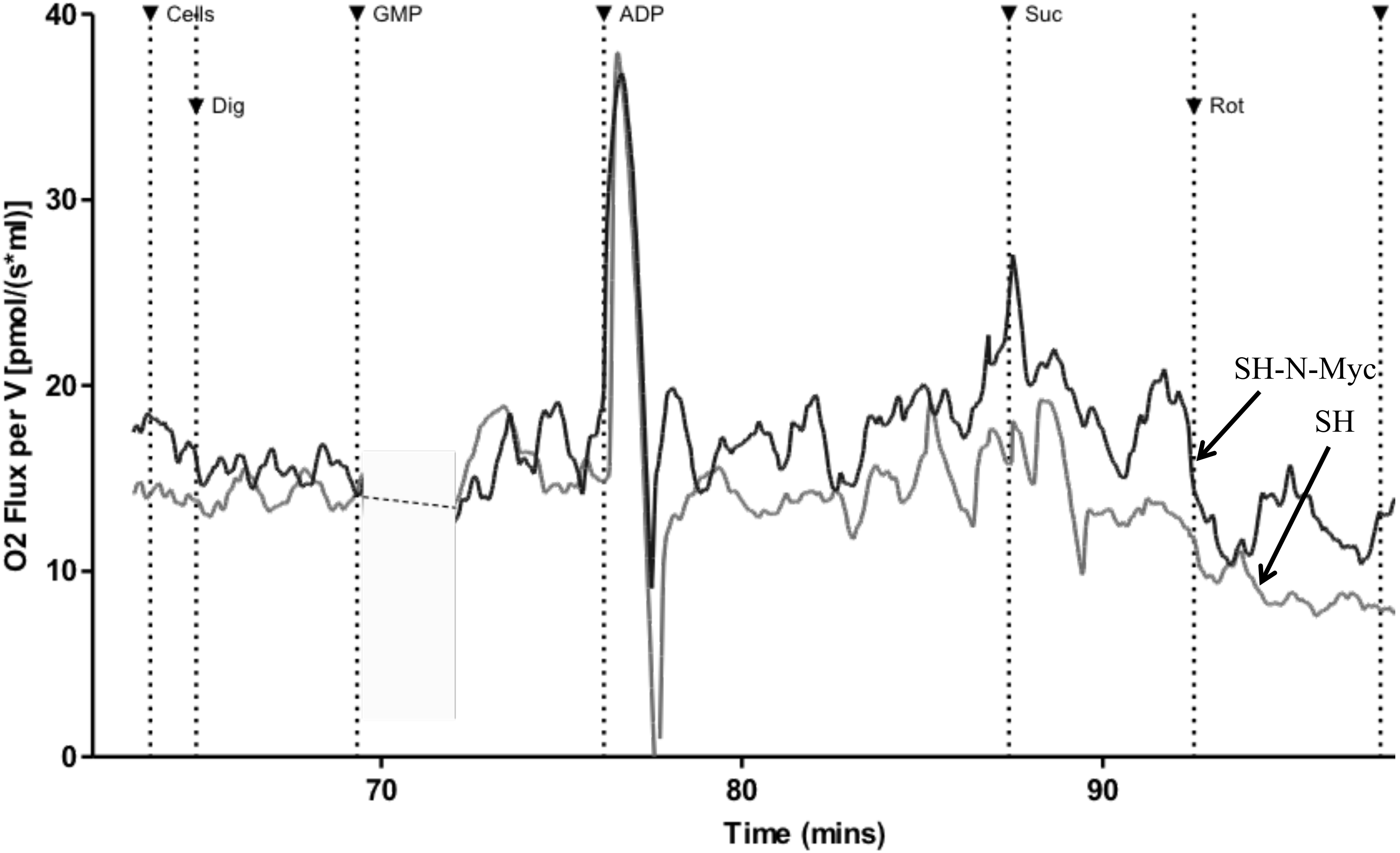

Supplemental Figure 5

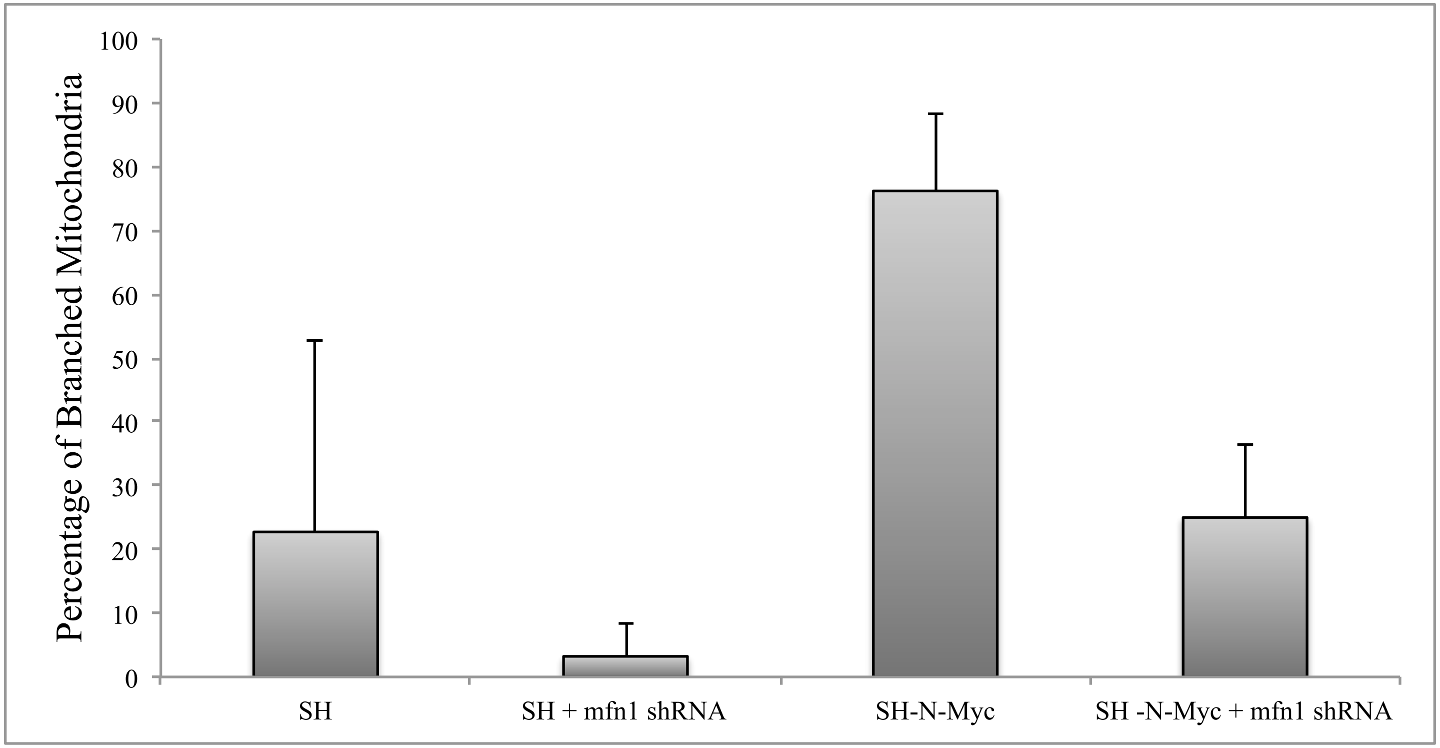

Supplemental Figure 6

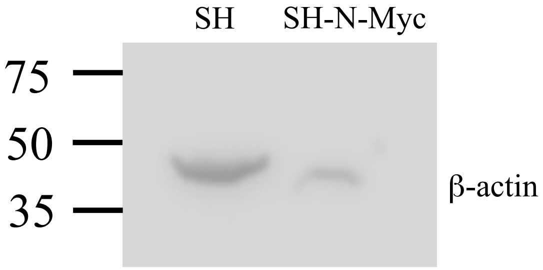

Supplemental Figure 7

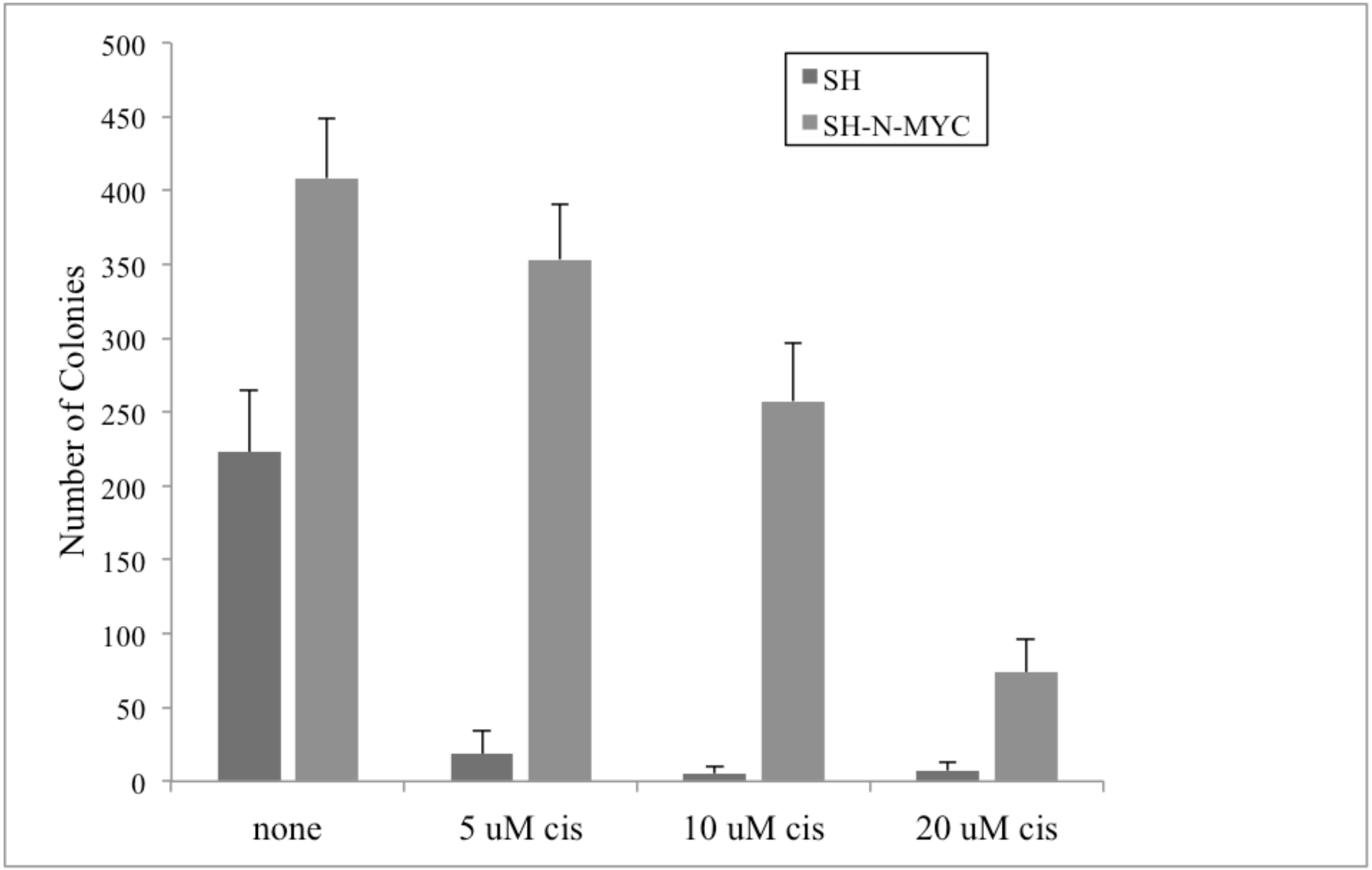

Supplemental Figure 8

Supplemental Figure 1. Western blot of WCL of BE2 showed N-Myc overexpression. Control sample was from SH cells.

Supplemental Figure 2. Quantification of MitoTracker Green fluorescence of SH and SH-N-Myc cells.

Supplemental Figure 3. Quantification of MitoTracker Deep Red fluorescence of SH and SH-N-Myc cells in the mitochondrial fusion assay.

Supplemental Figure 4. Cells were plated at 40-70% confluency onto glass bottom dishes and stained with JC-1 lives images were acquired by confocal microscopy. A red to green fluorescence ratio (R/G) was calculated, and a 50% increase was observed in SH-N-Myc cells when compared to controls.

Supplemental Figure 5. Identical numbers of cells in logarithmic phase were assayed on an Oroboros respirometer. Injections were done in the following order: 1. Cells, 2. Digoxin, 3. Glutamate, Malate, and Pyruvate (GMP), 4. ADP, 5. Succinate (Suc), 6. Rotenone (Rot).

Supplemental Figure 6. Cells were plated onto glass coverslips, stained with MitoTracker Deep Red then fixed and analyzed by confocal microscopy. The percentage of branched mitochondria were calculated for each cell type.

Supplemental Figure 7. Western blot of WCL of SH and SH-N-Myc cells detected no c-Myc protein. β-actin was used as a loading control.

Supplemental Figure 8. Clonogenic plate assay. Cells were exposed to media containing up to 20 μM cisplatin.
